# Military Personals

**Published:** 1918-08

**Authors:** 


### MILITARY PERSONALS

Col. J. G. Adami of Montreal, our associate editor for Canada, has completed the first volume of the history of the Canadian Army Medical Corps.

To Biltmore, N. C., Lieut. Lyman H. Wheeler, Lockport.

To Camp Dix, Lieut. Ira W. Livermore, Gowanda.

To Camp Jackson, S. C., Lieuts. Harold J. McDonald, Buffalo; Frank Walz, Buffalo.

To Camp Raritan, Metuchen, N. J., Lieut. Thos M. Calladine, Perry.

To Camp Wadsworth, Lieut. Harold Johnson, Friendship; Capt. Wm. F. Plumley, Rochester; Lieut. Lee Sachs, Buffalo.

To Camp Zachary Taylor, Louisville, Ky., Capt. Maurice B. Barnett, Watertown.

To Camp Sherman, O., Lieut. Carl G. Frost, Buffalo.

To Camp May, N. J., Capt. Lee M. Francis, Buffalo; Lieut. James C. Sullivan, Buffalo.

To Fort Leavenworth, Lieut. Daniel Jung, Buffalo.

To Fort Oglethorpe, Ga., Lieuts. Francis M. Kujawa, Buffalo; Francis J. Robinson, Rochester; Wm. W. Weglem, Rome; Herman E. Oak, S. Onondaga.

To Camp Meade, Lieut. Frederick Schenkelberger, Gowanda.

To Fort Porter, N. Y., Capt. Clarence H. Mackay, Lancaster.

To New Haven, Conn., Lieut. James H. Stygall, Buffalo.

To Newport News, Lieut. Abraham Silverman, Syracuse.

To Rockefeller Institute, Lieut. Wm. J. Galvin, Oswego.

To Washington, Lieut. Benjamin Slater, Rochester.

To Camp Gordon, Lieut. Joseph Zimmerman, Buffalo.

To Fort Sam Houston, Lieut. Ralph Gregorius, Corning.

To New York City, Lieut. Julius T. Cohen, Buffalo.

To Walter Reed General Hospital, Capt. Frederick Filsinger, Buffalo.

To Camp Devens, Lieuts. Myron Thompson, Albert R. Ellison, Buffalo.

To Fort Bliss, Capt. Wm. Woodbury, Rochester.

To Plattsburg Barracks, Capt. James Munson, Sonyea.

Capt. A. L. Benedict, of the editorial staff of this journal, has been transferred from Camp Greenleaf, Ga., to the Gas Defense Plant, Long Island City. He, with various other soldiers, enjoyed the hospitality of the Bristol (Va.) Red Cross Chapter in passing through that city.

